# Surgical Papers of the Transactions of the American Medical Association, 1870

**Published:** 1871-01

**Authors:** E. Andrews

**Affiliations:** 81 Monroe St., Chicago


Surgical Papers of the Transactions of the American Medical Association: 1870.

The 21st volume of this work appears earlier than usual, and in excellent style. The surgical papers are of excellent quality and well worthy of perusal. The first is

Median Lithotomy; by James Little, M.D., of New York.

Median Lithotomy. The principal interest of the paper centres in its large tabular list of cases, and their results. The reader of course will remember that median lithotomy is a modification of the old Marian operation, and consists in cutting into the membranous portion of the urethra upon a grooved staff, (making the incision in the median line), and dilating the neck of the bladder gently with the finger. The forceps are then introduced and the stone extracted; if too large, it is first crushed. Dr. Little reports 96 American cases of this operation. One of these should be excluded from the list, having been unjustly performed on a person already past hope. Of the remaining 95 cases, only 2 died, being one death in 47½ cases, which is certainly a far better record than the lateral operation can show, which, in Europe, has a mortality of one in five, and, in America, of one in sixteen.

New Operation for Imperforate Anus; by Thomas A. Healy, M.D., of Maryland.

This is a report of an operation performed, not by any means for an imperforate anus, as the title calls it, but for an anus of small size, situated too far forward. The operation consisted in dissecting around the anus and lower portion of the rectum, cutting a place for it further back, and sliding it along, and stitching it to its proper position. The result seems to have been good.

Form of Neuralgia of the Jaw-bone, Hitherto Undescribed; by Prof. S. D. Gross, of Philadelphia.

This disease consists of a neuralgia, caused by the gradual change in the alveolar processes after the loss of teeth, by which the extremities of the dental nerves become involved in the contraction of the bony canals in which they lie, so as to be pinched, as it were, and thus rendered permanently neuralgic. Dr. Gross cured them by dissecting away the gums, and with cutting bone forceps, cutting away the alveolar processes to the very base. Five cases are detailed, all of which were successful.

Partial Paralysis from Reflex Irritation, Caused by Congenital Phymosis and Adherent Prepuce; by Lewis A. Sayre, of New York.

The paper bearing the above title is a report of a few cases in which partial paralysis existed in cases of very tight congenital phymosis, with great irritability of the glans and meatus. A rapid cure resulted in each case, after circumcision and peeling up the adherent prepuce. This observation of Dr. Sayre is certainly striking, and worthy of further attention, to see if the experience of others will confirm his ideas.

Liquid for the Preservation of Wet Anatomical Preparations; by B. Titcomb, of Maryland.

This document gives a formula for a preserving fluid which may be usefully employed, but contains nothing new in principle. It preserves specimens and even entire subjects for dissection. The following is the formula:

℞ Chloride of sodium, ℥ iss.
Sulphate of alumina (doubtless meaning sulphate of alumina and potassa, or common alum), ℥ iss.
Nitrate of potassa, ʒ vj.
Water, s ℥ xij.

Preparatory to being placed in this fluid, the specimen is immersed in pure water 12 hours, and then in creasote water 12 hours more.

This solution will doubtless keep specimens perfectly as I have long ago proved by experiments with similar mixtures; but it ought properly to be stated that either the salt or alum alone will do the same thing. The creasote might quite as well, aud at a much cheaper rate, be supplanted by carbolic acid. The only use of the nitrate of potassa is to preserve something of the red color of the muscles. A solution of one part of alcohol, eight parts of water, and five grains to the ounce of carbolic acid is often used to preserve specimens in the museum of the Chicago Academy of Sciences.

Case of Congenital Occlusion of the Rima Glottidis; by Louis Elsberg, A.M., M.D., of New York.

This volume of the Transactions seems to abound in the erroneous use of terms by the authors. Above we noticed a paper on an "imperforate" anus, which was not imperforate, but only narrowed and misplaced, and now we have an "occlusion" of a rima glottidis, which is not occluded, (closed up), but only diminished in size by the adhesion of most of the anterior portion of the edges to each other. It was relieved by excising a portion of the obstructing membrane.

New Method of Reducing Dislocation at the Shoulder-joint; by Samuel Logan, M.D., of New Orleans.

This method consists in laying the patient supine upon the floor, while the surgeon sits at the same level opposite the afflicted shoulder, or a little toward the feet, at such a distance that his feet will just reach the patient's body. Taking the injured arm by the wrist, the surgeon places one heel just below the axilla, taking pains not to press the head of the humerous at all with his heel, while the rest of his foot, a little everted, rests against the ribs. The surgeon then places the ball of the great toe of the other foot against the acromian process above the shoulder, taking pains not to encroach too much with the foot upon the cavity of the joint. In this position he begins to make extension, at first a little downwards, and then squarely outwards, about at right angles to the line of the patient's body. If there is difficulty in accomplishing the reduction, the arm is brought downwards towards the feet, and pried as a lever across the heel, so as to throw the head of the bone into the joint.

A Contribution to Plastic Surgery; by Gurdon Buck, M.D., of New York.

This describes a case of deformity resulting from mortification of the right upper lip, cheek, and ala of the nose. Dr. Buck, by a series of four ingenious operations, removed most of the deformity; but the various steps are too numerous to be detailed here. The paper is illustrated by four beautiful colored lithographs. There is no absolutely new principle introduced, but the series of operations constitute a fine study in plastic surgery.

Case of Formation of Bone in the Eye; by Chas. M. Carleton, of Conn.

This paper is a report of a case where a patient was attacked with neuralgia of the right eye and total loss of its vision. After a time it was extirpated, when between the retina and the choroid there was found a "large, irregular, cup-shaped formation of true bone."

A New Mode of Amputation at the Ankle-joint; by J. N. Quimby, of New Jersey.

This is a relation of cases of amputation at the ankle by a modification of Pirogoff's operation. His incisions of the soft parts are made after the manner of Pirogoff, but his next step is to dissect out the astragulus, and saw off the anterior portion of the *os calcis* obliquely, downward and forward. Next, without removing any of the *tibia* nor *fibula*, nor even the cartilage, the cut end of the *os calcis* is placed against the articular surface of the *tibia* and retained by sutures and adhesive straps. The wounds healed by first intention in a very short time, making stumps on which the patient walked with ease. I incline to think this modification is worthy of further trial.

A New Method of Lithotrity; by E. M. Moore, M.D., of Roch ester, N. Y.

This a paper which, though brief, has a good deal of merit. Dr. Moore proposes to complete the operation of lithotrity and remove all the fragments at cne sitting. The description of his instruments is so brief that it is impossible to get a clear idea of them; the principle, however, is this: A steel catheter carries a folded steel net, which, when inserted into the bladder, can be expanded, and made to surround the stone and draw it against the end of the instrument. A scraping instrument then cuts the stone to pieces and allows of all the debris being brought away at one sitting. He reports one case which was highly successful. The instrument must be extremely curious and ingenious.

On the whole, this volume presents a very creditable surgical array, and is, perhaps, in that respect better than any of its predecessors.

Prize Essay on the Treatment of Aneurism: by Benjamin Howard, A.M., M.D., New York.

In noticing the surgical papers of the transactions previously handed you, this paper was accidentally omitted.

It is one of very considerable merit, being an original experimental investigation into the effects of a new method of ligating arteries for aneurism.

Part first consists of an examination of the nature and effects of atheromatous deposits in producing aneurism. The author considers atheroma as not a growth from the walls of the vessels, but a deposit from the blood within it.

This deposit consists of granules, oily globules, and cholestrine, and exists as elevated patches on the inner surface of the vessels. Where the patch is attached, the coats of the artery undergo atrophy, and the patch itself degenerates in various ways. After a time the weakened spot in the vessel yields to the force of the blood and dilates into an aneurism.

He then goes on to maintain, that when the blood in an aneurism is suddenly stopped in its motion by a tight ligature, it coagulates into a black clot, which is essentially a dead, foreign body, and must be either absorbed or excite inflammation and suppuration, and thus be cast out. On the contrary, if the flow of the blood is simply diminished, the aneurism is gradually filled with the white, or rather the buff clot, which becomes permanently organized, and excites no suppuration or inflammation.

To illustrate this subject he performed thirteen operations upon the caroted arteries of sheep. Three of these consisted in tying the common carotid artery with silver and lead ligatures tightly closed, so as to completely arrest the flow of blood. The wires were cut off close and left in the wound. The incisions all healed by first intention, but after a month or two, a dissection always showed the formation of an abscess containing the ligature, and the clot was imperfect.

Ten of the experiments consisted in tying the artery with metalic ligatures (usually silver wire) applied loosely, so as only to obliterate from half to two-thirds of the calibre of the artery. The ligatures were cut off close, and the wounds healed over them by first intention, as before. In these cases the arteries became occluded as before, but there was no inflammation and no abscess, though some of the ligatures were left in the flesh two years. The clot, also, was better organized, and all the appearances more perfect than in the former cases. The experiments are illustrated by eighteen excellent lithographs.

This method of ligation would seem to promise much better results than the old methods, and is certainly worthy a fair trial by the surgeon.

Any investigation tending to diminish the mortality of ligations of large vessels, is of great importance, for, notwithstanding the fasinating beauty of the operation from a mere surgical point of view, there is a heavy mortality accompanying it. The average deathrate from ligatures of all the large arteries, is over thirty-three per cent., and ligatures of the femoral artery are more dangerous than amputations of the thigh.

E. ANDREWS, M.D.,
81 Monroe St., Chicago.

